# Postepidemic Analysis of Rift Valley Fever Virus Transmission in Northeastern Kenya: A Village Cohort Study

**DOI:** 10.1371/journal.pntd.0001265

**Published:** 2011-08-16

**Authors:** A. Desirée LaBeaud, Samuel Muiruri, Laura J. Sutherland, Saidi Dahir, Ginny Gildengorin, John Morrill, Eric M. Muchiri, Clarence J. Peters, Charles H. King

**Affiliations:** 1 Center for Immunobiology and Vaccine Development, Children's Hospital Oakland Research Institute, Oakland, California, United States of America; 2 Center for Global Health and Diseases, Case Western Reserve University, Cleveland, Ohio, United States of America; 3 Division of Vector-Borne and Neglected Tropical Diseases, Ministry of Public Health and Sanitation, Nairobi, Kenya; 4 Center for Biodefense and Emerging Infectious Diseases, University of Texas Medical Branch, Galveston, Texas, United States of America; University of Texas Medical Branch at Galveston, United States of America

## Abstract

**Background:**

In endemic areas, Rift Valley fever virus (RVFV) is a significant threat to both human and animal health. Goals of this study were to measure human anti-RVFV seroprevalence in a high-risk area following the 2006–2007 Kenyan Rift Valley Fever (RVF) epidemic, to identify risk factors for interval seroconversion, and to monitor individuals previously exposed to RVFV in order to document the persistence of their anti-RVFV antibodies.

**Methodology/Findings:**

We conducted a village cohort study in Ijara District, Northeastern Province, Kenya. One hundred two individuals tested for RVFV exposure before the 2006–2007 RVF outbreak were restudied to determine interval anti-RVFV seroconversion and persistence of humoral immunity since 2006. Ninety-two additional subjects were enrolled from randomly selected households to help identify risk factors for current seropositivity. Overall, 44/194 or 23% (CI_95%_:17%–29%) of local residents were RVFV seropositive. 1/85 at-risk individuals restudied in the follow-up cohort had seroconverted since early 2006. 27/92 (29%, CI_95%_: 20%–39%) of newly tested individuals were seropositive. All 13 individuals with positive titers (by plaque reduction neutralization testing (PRNT_80_)) in 2006 remained positive in 2009. After adjustment in multivariable logistic models, age, village, and drinking raw milk were significantly associated with RVFV seropositivity. Visual impairment (defined as ≤20/80) was much more likely in the RVFV-seropositive group (*P*<0.0001).

**Conclusions:**

Our results highlight significant variability in RVFV exposure in two neighboring villages having very similar climate, terrain, and insect density. Among those with previous exposure, RVFV titers remained at >1∶40 for more than 3 years. In concordance with previous studies, residents of the more rural village were more likely to be seropositive and RVFV seropositivity was associated with poor visual acuity. Raw milk consumption was strongly associated with RVFV exposure, which may represent an important new focus for public health education during future RVF outbreaks.

## Author Summary

RVFV infection causes significant disease in both human and animal populations, resulting in significant agricultural, economic and public health consequences. We conducted a cohort study on residents of a high-risk area to measure human anti-RVFV seroprevalence, to identify risk factors, and to estimate the durability of prior RVFV immunity. One hundred two individuals tested for RVFV exposure before the 2006–2007 RVF outbreak were restudied to determine interval anti-RVFV seroconversion and persistence of humoral immunity since 2006. Ninety-two additional subjects were enrolled from randomly selected households to help identify risk factors for current seropositivity. Seroprevalence in the region was high (23%). 1/85 at-risk individuals restudied in the follow-up cohort had seroconverted since early 2006. 29% of newly tested individuals were seropositive. After adjustment in multivariable logistic models, age, village, and drinking raw milk were significantly associated with RVFV seropositivity. Visual impairment (defined as ≤20/80) was much more likely in the RVFV-seropositive group. Among those with previous exposure, RVFV titers remained at protective levels (>1∶40) for more than 3 years. This study highlights the high seroprevalence among Northeastern Kenyans and the ongoing surge in seroprevalence with each RVF outbreak.

## Introduction

Rift Valley Fever (RVF) is a life-threatening, mosquito-borne zoonotic disease found in many areas of sub-Saharan Africa and the Middle East [1]. Because Rift Valley fever virus (RVFV) readily infects both humans and their livestock, RVF poses a severe, dual threat to public health and to livestock food production in endemic regions [2], [3]. Of particular concern, the range of RVFV transmission has extended beyond sub-Saharan Africa over the last 35 years [4]–[6]. Future RVFV spread beyond its present enzootic areas, whether through natural livestock/vector movement or through bioterrorist action, poses a significant threat to many countries. RVFV, a member of the genus *Phlebovirus*, is a mosquito-borne virus that is maintained within ecosystems by vertical transmission among local floodwater *Aedes* spp. mosquitoes [7]. Typically, in enzootic regions, these transient vectors reintroduce RVFV into local mammalian fauna following periods of heavy rainfall, after which other hematophagous vectors, typically culicine mosquitoes, serve to perpetuate transmission [8]. In addition, transmission of RVFV can also occur via aerosol or direct contact with infected animals or their body fluids [9].

RVFV infection causes serious disease in both human and animal populations, resulting in significant agricultural, economic and public health consequences. Although in the majority of human cases RVFV causes a mild, acute febrile illness with fever, malaise, and myalgia, a minority of human cases are complicated by retinitis (10%), encephalitis (8%), and hemorrhagic fever (1%) with significant risk of related morbidity and mortality [9]–[18]. During outbreaks, livestock are at even greater risk, as RVF frequently causes hemorrhagic disease and “abortion storms” that are associated with high mortality among domestic sheep, goats, and cattle [3], [19], [20].

In 2006–2007, a major Rift Valley Fever outbreak resulted in significant human and animal disease across East Africa, including parts of Kenya, Tanzania, Sudan, and Somalia [21], [22]. In Kenya, 684 human cases were reported of whom 333 were from Northeastern Province, the focus of our present study [21]. Having conducted a serosurvey in Ijara District in 2006, just prior to the 2006–2007 outbreak, we then performed a follow-up survey in 2009 in order to: (i) quantify the new local level of anti-RVFV seroprevalence in the human population; (ii) identify risk for seroconversion; and (iii) monitor previously exposed individuals to estimate the persistence of their post-infection immune response.

## Methods

### Ethics Statement

All adult participants provided written informed consent under a protocol approved by the Human Investigations Review Board of University Hospitals of Cleveland and by the Ethical Review Committee of the Kenya Medical Research Institute, Nairobi. Parents provided written informed consent for participating children; children >7 years of age also provided individual assent.

### Location

**Figure 1:**
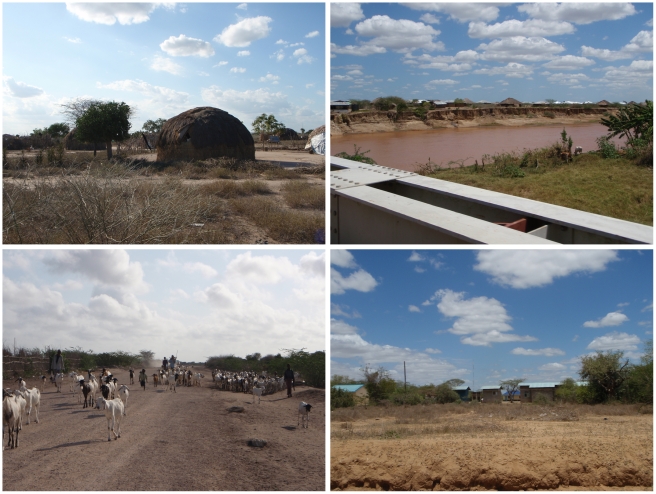
Pictures of Masalani. Left upper, Gumarey homestead; Left lower, local herd; Right upper, Sogan-Godud homestead; Right lower, view of Masalani town and Tana River from Masalani bridge.

Our study was a household-based cluster sampling of human populations residing in 2 areas near Masalani Town, Ijara District, situated in a semiarid region of Northeastern Province, Kenya (Figure 1).

The study was performed in August through November of 2009, ∼3 years after the previous RVF outbreak of 2006–2007 [22]. This population was previously tested for RVFV in early 2006 [23] prior to the latest major RVF outbreak, and was revisited to re-enroll previous participants in order to monitor incidence and anti-RVFV IgG seroprevalence changes since the last outbreak. The participants were selected from two villages: a rural village, Gumarey (centered at 1°40′12″S, 40°0′48″E), and a town, Sogan-Godud (centered at 1°41′24″S, 40°10′12″E). Both are sublocations of the Masalani Division of Ijara District as defined within the Kenya Census, and both have suffered repeated RVF outbreaks, most recently in 1997–1998 [24] and 2006–2007 [22]. Gumarey is more rural having a predominantly semi-nomadic pastoralist population. Sogan-Godud is a larger town with a central marketplace with a more permanent population (Figure 1).

### Objectives

Participants were either newly enrolled or re-enrolled for anti-RVFV IgG testing following a RVF epidemic/epizootic in Masalani Town, Northeastern Province. Sera were tested for the presence of anti-RVFV IgG antibodies by standardized testing (indicative of previous exposure to virus infection). The follow-up specimens were obtained to identify seroconversions following a known RVF outbreak and to investigate epidemiologic risk factors that were related to current seropositivity. For those who had been seropositive in 2006, we also wished to estimate the temporal duration of RVF antibody positivity. Given the extensive nature of the 2006–2007 RVF outbreak in this region and the longstanding history of RVF in Kenya, we hypothesized that we would be able to identify >13% of individuals in these villages as seropositive for anti-RVFV antibodies, and that our follow-up cohort would contain seroconverters after the outbreak. We conducted an extensive behavioral survey to elicit a greater understanding of risk-related RVFV exposure factors and to identify risk behaviors that might be relevant for targeted public health activities.

### Participants

Study recruitment began after consultation and approval by local leaders and administrators. After an initial demographic census was performed to determine the current local population and its distribution, up to three attempts were made to contact and re-enroll each participant from our previous serosurvey [23]. Another group of new survey participants were randomly selected by household clusters in the 2 designated villages of Masalani town. Those living in the area less than 2 years and children more than 1 year of age were excluded. The study sample was representative of the local ethnic mix of >99% Somali or Bantu and <1% Indian or other Asian. Participating households were sampled by using a probability of selection proportionate to size approach. Households were randomly selected until we reached our sample size goal of 200 enrolled individuals.

### Examination Procedures

Study participants each received a formal interview detailing demographics, occupation, housing, mosquito exposure, animal exposure, motor ability, visual ability, and recent or remote RVF-related symptoms (questionnaire in Appendix S1). When necessary, accompanying parents served as proxies in answering for younger children. Each subject also received a complete physical examination, vision testing, and indirect ophthalmoscopic examination for signs of current or previous retinal inflammation. Serology testing was performed on specimens obtained by same-day phlebotomy (i.e., venous blood samples 5 ml in those ≥5 years of age and 1 ml in children <5 years of age).

### Laboratory Testing

RVFV exposure was initially determined by serum anti-RVFV IgG detection using ELISA [23], [25], [26] and confirmed by plaque reduction neutralization testing (PRNT_80_) [27], [28]. A PRNT_80_ titer of >1∶20 was considered positive. Specimens having an ELISA OD value of >0.25 were considered positive. Briefly, specimens were initially screened for the presence of anti-RVFV IgG by ELISA by using lysates of Vero cells infected with the MP-12 strain (vaccine strain [27]) of RVFV as the test antigen and lysates of mock-infected cells as the internal control antigen, as established and validated in previous survey studies [23], [25], [26]. Confirmatory plaque reduction neutralization testing (PRNT_80_) was performed at University of Texas, Medical Branch at Galveston to assess the risk of false-positive results secondary to ELISA cross-reaction with related viruses. This confirmatory testing using PRNT_80_ was performed on all positive samples (n = 44) and a set of borderline negative samples (n = 25) [27]. ELISA testing revealed incongruent results with PRNT_80_. Five ELISA-positive samples were negative by PRNT_80_ (titers<1∶20). Ten ELISA-negative samples had titers ≥1∶20. Most ELISA positive samples had PRNT_80_ titers of 1∶320. Overall, ELISA had 77% sensitivity, 97% specificity, 87% positive predictive value, and 94% negative predictive value when compared to gold standard PRNT_80_. All anti-RVFV serology results discussed in this manuscript are based on PRNT_80_ results.

### Statistical Methods

Summary statistics were computed to describe demographic variables. The primary outcome was RVFV seropositivity as determined by PRNT_80_. Bivariate analysis was based on χ2 tests (or Yates' correction to the χ2 where appropriate) of potential categorical predictors of seropositivity as well as bivariate comparisons between villages. Independent t-tests were used for bivariate comparisons of continuous predictors. The multivariable logistic regression models used for estimation of the adjusted odds ratios for seropositivity utilized data from all 194 participants. These models were initially developed using predictor variables that had been determined in bivariate analysis to be significantly associated with RVFV seropositivity. In addition, separate models were constructed using only those subjects who were repeat survey participants (N = 102) or using only those who were new participants (N = 92). Logistic models were also constructed separately for each village in order to determine significant local predictors of RVFV seropositivity. A collinearity analysis was performed examining all of the potential predictors in the models [29]; however, no evidence of collinearity was found. Hosmer-Lemeshow goodness-of-fit χ2 square tests were calculated for all logistic models and indicated that model predictors sufficiently described the observed data. All bivariate analysis and logistic modeling was performed using SAS software (SAS Institute Inc., version 9.2, Cary, NC, USA). A significance level of 0.05 was used for all statistical tests.

## Results

### Survey results: seroprevalence by group and seroconversion

**Figure 2:**
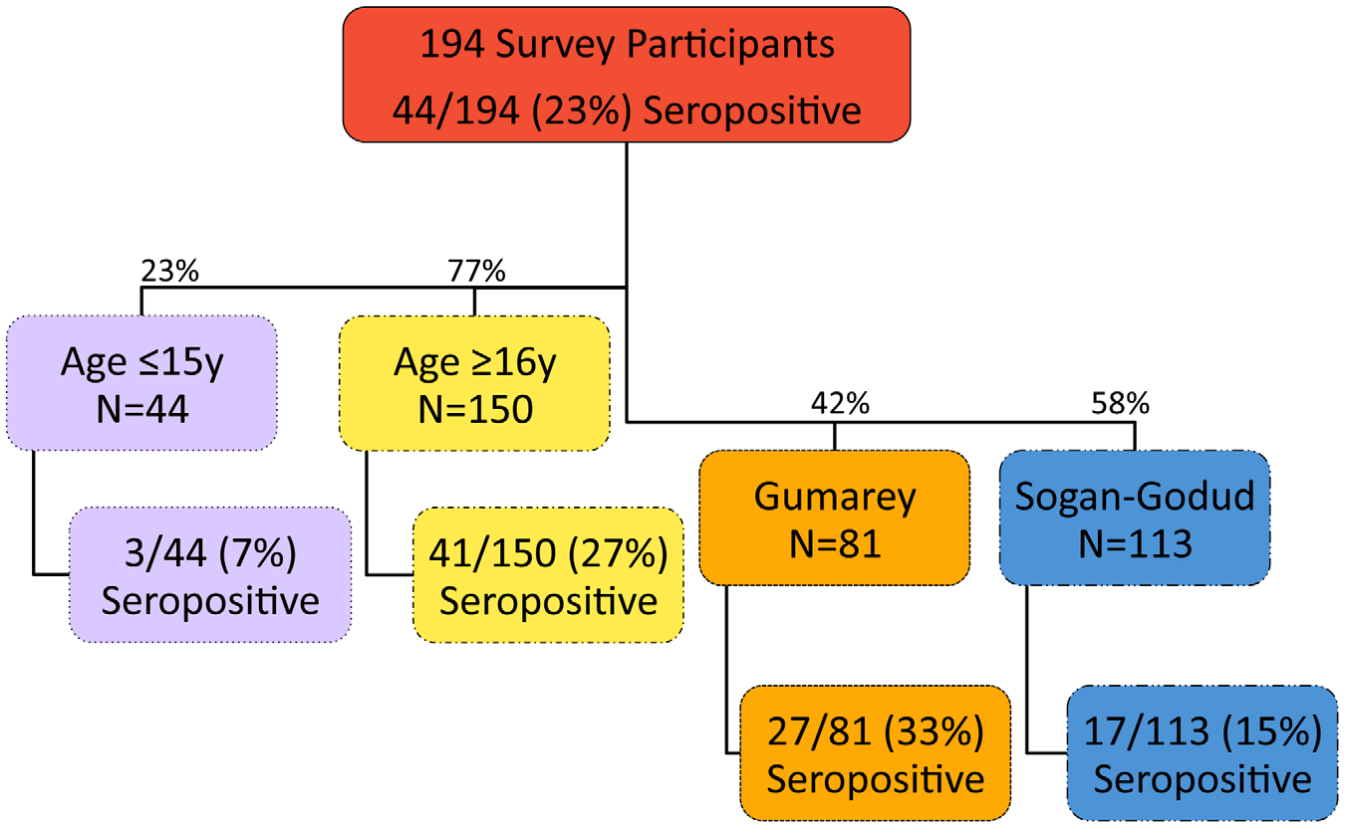
Flow chart of study samples.

A total of 194 participants were enrolled in this study: 102 had participated in the previous serosurvey [23] and 92 were new participants (Table 1). Of the total 194 participants, 44 were RVFV seropositive (23%, CI_95%_: 17%–29%). Among all participants, 81 (42%) were from the more rural village, Gumarey (GM, from 45 households) and of these, 27 (33%, CI_95%_: 23%–44%) were seropositive (See Figure 2). The remaining 113 subjects (58%) were from the more developed area, Sogan-Godud (SG, from 64 households) and of these, 17 (15%, CI_95%_: 9%–23%) were seropositive. Of all samples, 44 (23%) were from children ≤15 years of age, of whom 3 (7%, CI_95%_: 0%–14%) were seropositive. These 3 youngest seropositive participants were 7, 7, and 15 years of age, and all were long-time, permanent residents of the study area. Of the 150 adults sampled, 41 (27%, CI_95%_: 20%–34%) had positive anti-RVFV IgG results; the oldest was 82 years of age.

**Table 1:** Study demography and anti-Rift Valley fever virus serology results by sex, age group, and village.

	RepeatSubjects(N = 102)	NewSubjects(N = 92)	AllSubjects(N = 194)
**Sex**			
Female	66 (65%)	57 (62%)	123 (63%)
Male	36 (35%)	35 (38%)	71 (37%)
**Age**			
Adults	67 (66%)	83 (90%)	150 (77%)
Children (≤15 years)	35 (34%)	9 (10%)	44 (23%)
**Village**			
Sogan-Godud	60 (59%)	53 (58%)	113 (58%)
Gumarey	42 (41%)	39 (42%)	81 (42%)
**RVFV Seropositives**			
Both Villages	17 (17%)	27 (29%)	43 (22%)
Sogan-Godud	6 (7%)	11 (12%)	16 (8%)
Gumarey	11 (12%)	16 (17%)	27 (14%)

**Figure 3:**
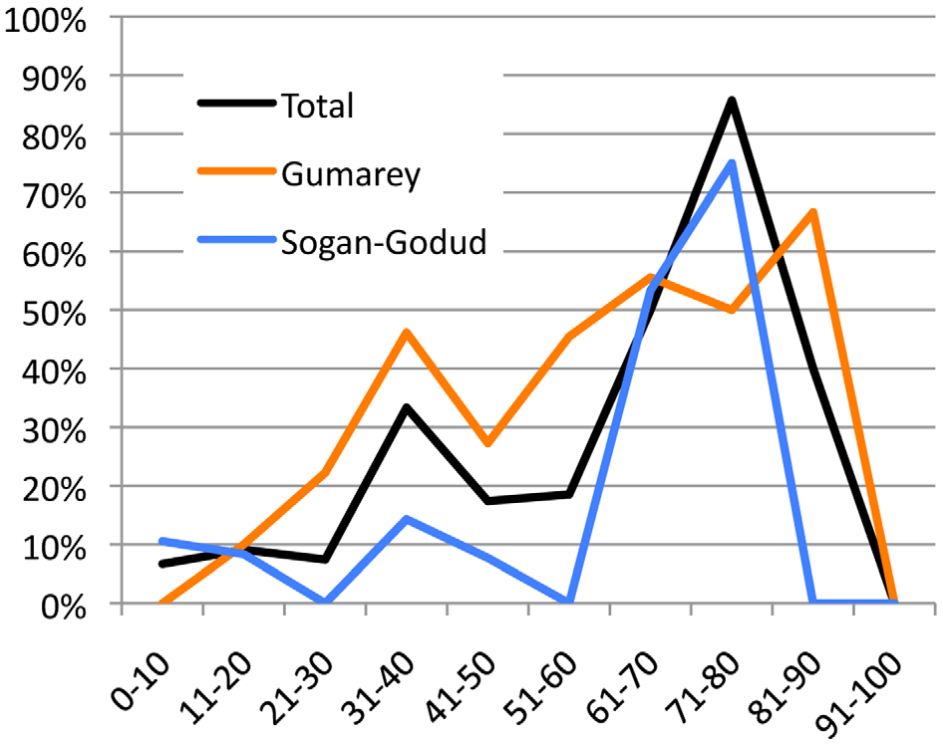
Seroprevalence of age groups from study villages and total sample.

One 77 y/o female from SG seroconverted in the interval between testing in early 2006 and August 2009 [23]. Twenty-six newly tested individuals were seropositive (28%, CI_95%_: 20%–39%). Participants from Gumarey were more likely to be RVFV seropositive in nearly every age group (see Figure 3).

New participants were more likely to be RVFV seropositive (*P*<0.001) and have impaired visual acuity (worse than 20/40) during eye examination (*P* = 0.009) (Table 2). Comparison of the new and repeat study participants demonstrated that new participants were more likely to be older, nomadic, live in semi-permanent housing, have recent home flooding, use mosquito nets, use fire as mosquito control, have ill family members, have dead body contact, have dirt flooring and have sheep and camel contact. New participants were also more likely to report recent symptoms of illness. New participants were less likely to report specific animal exposures when compared to repeat participants.

**Table 2:** Comparison of new and repeat participants.

	New Participants	Repeat Participants	P value*
	(N = 92)	(N = 102)	
RVFV seropositive	28%	18%	<0.0001
Poor visual acuity during eye exam	62%	43%	0.009
Age, y: Mean + SD	44.9±21.0	33.2±22.6	0.0003
Nomadic	89%	25%	<0.001
Live in semi-permanent housing	70%	21%	<0.001
Have recent home flooding	7%	31%,	<0.001
Use mosquito nets	93%	95%,	<0.001
Use fire as mosquito control	97%	88%	<0.001
Have ill family members	73%	94%	<0.001
Have dead body contact	14%	43%	<0.001
Have dirt flooring	88%	57%	<0.001
**Animal Exposures**			
Sheep contact	61%	71%	<0.001
Camel contact	29%	9%	<0.001
Sheltering livestock	21%	61%	<0.001
Killing livestock	11%	30%	<0.001
Butchering livestock	20%	68%	<0.001
Milking livestock	32%	71%	<0.001
**Reported recent symptoms of illness**			
Myalgia	52%	28%	<0.001
Eye pain	69%	20%	<0.001
Headache	86%	53%	<0.001
Red eyes	78%	33%	<0.001
No appetite	72%	29%	<0.001
Photophobia	60%	24%	<0.001
Vertigo	33%	16%	<0.001
Stupor	24%	4%	<0.001
Meningismus	46%	14%	<0.001
Poor vision	47%	19%	<0.001

*Calculated by chi-square testing with Yates' correction, as appropriate.

### Repeat PRNT_80_ testing

**Figure 4:**
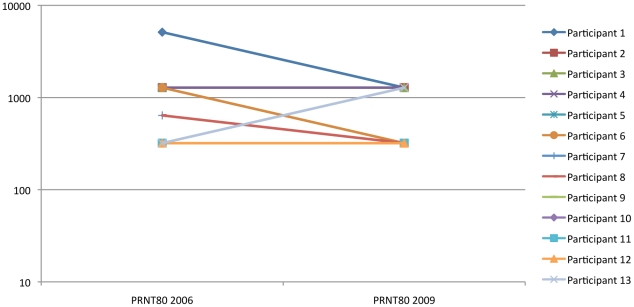
Plaque Reduction Neutralization Titer (PRNT_80_) of 13 individuals with repeated testing.

Thirteen individuals who were confirmed anti-RVFV positive by PRNT_80_ in the prior 2006 survey [23] had repeat PRNT_80_ performed in the current study (see Figure 4). All had positive titers ≥1∶ 320 in 2009. Seven subjects had titers that remained unchanged. Five had a 1- to 4-fold drop in titer. One individual had a two-fold boost in titer from 1∶320 to 1∶1280.

### Links between past exposures and seropositivity

Many exposures, both non-animal and animal, were associated with RVFV seropositivity (Table 3). In bivariate statistical analyses, RVFV seropositivity varied significantly according to the following factors: age (participants >15 years of age were more at risk, OR 0.20, CI_95%_: 0.06–0.66, *P*<0.001), gender (male participants were more at risk, OR 2.33, CI_95%_: 1.18–4.61, *P* = 0.020), location (those from Gumarey were more at risk, OR 2.82, CI_95%_: 1.41–5.64, *P* = 0.003), drinking raw milk (OR 2.71, CI_95%_: 1.36–5.42, *P* = 0.005), and involvement in skinning livestock (OR 2.12, CI_95%_: 1.06–4.24, *P* = 0.043), birthing livestock (OR 3.62, CI_95%_:1.61–8.15, *P* = 0.002), or disposing of an aborted animal fetus (OR 3.49, CI_95%_:1.52–7.99, *P* = 0.004).

**Table 3:** Bivariate analysis of anti-RVFV seropositivity according to demographic and exposure factors.

Variable*	P value†	Odds ratio(95% Confidence Interval)
Age (continuous)	<0.001	1.04 (1.02, 1.06)
Location (Gumarey vs. Sogan-Godud)	0.003	2.82 (1.41, 5.64)
Gender (Male vs. female)	0.020	2.33 (1.18, 4.61)
Shelter cow	0.006	3.81 (1.54, 9.38)
Kill cow	0.002	6.40 (1.98, 20.73)
Skin animal	0.043	2.12 (1.06, 4.24)
Skin cow	0.038	2.42 (1.07, 5.46)
Drank raw milk	0.005	2.71 (1.36, 5.42)
Drank raw sheep milk	0.035	2.11 (1.07, 4.16)
Drank raw cow milk	0.043	2.12 (1.06, 4.24)
Assist with birthing livestock	0.002	3.62 (1.61, 8.15)
Assist with birthing sheep	0.007	3.35 (1.43, 7.85)
Assist with birthing goat	0.007	3.35 (1.43, 7.85)
Assist with birthing cow	<0.001	8.47 (2.72, 26.40)
Dispose of aborted animal fetus	0.004	3.49 (1.52, 7.99)
Dispose of aborted sheep fetus	0.005	3.62 (1.53, 8.56)
Dispose of aborted goat fetus	0.021	2.98 (1.25, 7.08)
Dispose of aborted cow fetus	0.007	4.51 (1.53, 13.25)
Recent malaise	0.039	2.47 (1.07, 5.73)
Recent backache	0.014	2.57 (1.20, 5.48)
Recent rash	0.009	3.85 (1.48, 10.00)
Recent confusion	0.015	4.26 (1.35, 13.44)
Recent stupor	0.006	3.40 (1.44, 8.06)
Recent bloody stool	0.013	5.41 (1.45, 20.15)
Anterior chamber disease	<0.001	N/A
Posterior chamber disease	0.006	3.45 (1.46, 8.15)
Retinal disease	0.033	3.73 (1.13, 12.31)
Abnormal eye exam	0.003	6.21 (1.91, 20.20)
Poor visual acuity (≤20/80)	<0.001	5.09 (2.28, 11.32)

*All variables were dichotomous except age (continuous).

**†:** Pearson χ^2^ test with Yates' continuity correction was used for all variables except age (continuous), which used independent samples 2-tailed t test.

Other reported exposures varied significantly between the 2 sublocation groups. Those from Gumarey were more likely to shelter livestock (OR 2.2, CI_95%_: 1.18–3.8, *P* = 0.013), kill livestock (OR 2.1, CI_95%_: 1.03–4.16, *P* = 0.049), or have an ill family member (1–2 years ago OR 4.0, CI95%: 0.32, 49.60; 1–3 months ago OR 1.04, CI95%: 0.44, 2.42; 4–6 months OR 5.33, CI95%: 1.60, 17.83; 7–12 months ago OR 10.00, CI95%: 1.03, 97.49; and less than 1month ago OR 2.00, CI95%: 0.41, 9.71, as compared to never having an ill family member *P* = 0.003) (Table 4).

**Table 4:** Bivariate analysis of demographic and other exposure factors for anti-RVFV seropositivity by village location.

Variable	P value‡	Odds ratio comparingGumarey vs. Sogan-Godud(95% C.I.)
**When ill family member (ordinal)**	0.003	
1–2 yrs		4.00 (0.32, 49.60)
1–3 months		1.04 (0.44, 2.42)
4–6 months		5.33 (1.60, 17.83)
7–12 months		10.00 (1.03, 97.49)
Less than 1 month		2.00 (0.41, 9.71)
Never (reference)		1.00
**Latrine type**	0.045	
VIP		0.60 (0.06, 6.08)
Bush		2.23 (1.12, 4.46)
Pit		0.90 (0.43, 1.90)
Toilet (reference)		1.00
**Shelter livestock in home**	0.013	2.19 (1.18, 3.80)
**Shelter sheep in home**	0.012	2.16 (1.20, 3.90)
**Shelter goat in home**	0.007	2.35 (1.30, 4.26)
**Shelter cow in home**	<0.001	6.11 (2.16, 17.27)
**Kill livestock**	0.049	2.07 (1.03, 4.16)
**Kill sheep**	0.047	2.08 (1.02, 4.24)
**Kill goat**	0.019	2.38 (1.16, 4.87)
**Kill cow**	0.017	5.12 (1.36, 19.24)
**Assist with sheep birth**	0.021	2.71 (1.17, 6.28)
**Assist with goat birth**	0.006	3.27 (1.39, 7.72)
**Assist with cow birth**	0.002	6.32 (1.72, 23.20)
**Dispose of sheep fetus**	0.011	3.04 (1.28, 7.23)
**Dispose of goat fetus**	0.011	3.04 (1.28, 7.23)
**Dispose of cow fetus**	0.014	4.24 (1.30, 13.84)
**Herder occupation**	0.058	2.97 (0.97, 9.07)
**Recent fever**	0.034	1.96 (1.06, 3.63)

The final logistic model to predict RVFV seropositivity included age, location, and drinking raw animal milk (Table 5). In multivariable models used to predict adjusted odds of RVFV seropositivity, location was significant when age and raw milk consumption were controlled for; those residing in GM were at 3 times the risk of those in SG (adjusted OR 3.33; CI_95%_: 1.53–7.21). After age and location were controlled for, those who had consumed raw milk were 3 times more likely to be seropositive (adjusted OR 2.9, CI_95%_: 1.34–6.27). Children ≤15 years of age had a much lower risk for RVFV seropositivity than those >15 years of age. The adjusted OR for seropositivity (calculated from the overall logistic model) was 1.04; (CI_95%_: 1.02–1.06) per year of age. This difference persisted at both sublocation levels with adults in SG or GM at significantly higher risk than children.

**Table 5:** Logistic Regression Analysis to predict Rift Valley fever virus seropositivity.

Predictor variable	Variable type	Adjusted OR (CI)	*P* value
Age	Continuous	1.04 (1.02–1.06)	<0.0001
Location (Gumarey vs. Sogan-Godud)	Dichotomous	3.3 (1.5–7.2)	0.003
Drank raw milk	Dichotomous	2.9 (1.3–6.3)	0.006

Logistic Model 1, all participants*.

*CI, 95% confidence interval. Goodness-of-fit: Hosmer and Lemeshow test, p value = 0.489; R^2^ = 28.6%.

Models between old and new survey participants differed (Table S1A and B). Whereas, after multivariable adjustment, older age and male gender had the most significant association with anti-RVFV seropositivity among repeat participants, a history of attending to a birthing animal was the most significant predictor for new participants.

Subgroup analysis by village showed the significant predictor of RVFV seropositivity in Sogan-Godud to be age, cooking meat, and drinking raw milk (Table S2A). Older participants had a 4.5% increase in odds for each year of age. Those who cooked meat were less likely to be seropositive (adjusted OR, 0.184, CI_95%_:0.042–0.81). Those who consumed raw milk were nearly 16 times more likely to be seropositive (adjusted OR 15.7, CI_95%_: 2.9–84.9). In Gumarey, the higher risk village, the logistic model to predict seropositivity included age, such that the odds of seropositivity increased 5% for every 1-year increase in age (adjusted OR 1.05, CI_95%_: 1.02–1.07)(Table S2B).

### Links between seropositivity and symptom history or abnormal physical findings

A past history of malaise (OR 2.5, CI_95%_: 1.5–5.7, *P* = 0.004), backache (OR 2.6, CI_95%_: 1.2–5.5, *P* = 0.014), rash (OR 3.9, CI_95%_: 1.5–10.0, *P* = 0.009), stupor (OR 3.4, CI_95%_: 1.4–8.1, *P* = 0.006), confusion (OR 4.3, CI_95%_: 1.3–13.4, *P* = 0.015), or bloody stools (OR 5.4, CI_95%_: 1.4–20.1, *P* = 0.013) was statistically associated with RVFV seropositivity in the study population. Upon physical examination, no non-ocular examination finding was specifically associated with RVFV seropositivity.

Regarding ocular findings, anterior and posterior chamber abnormalities were associated with RVFV seropositivity: those with abnormal eye exam (OR = 6.2, CI_95%_: 1.9–20.2, *P* = 0.002), poor visual acuity (defined as ≤20/80; OR = 5.1, CI_95%_: 2.3–11.4, *P*<0.001), anterior chamber disease (*P*<0.001), posterior chamber disease (OR = 3.4, CI_95%_: 1.5–8.2, *P* = 0.006), and retinal disease (3.73, CI_95%_: 1.13–12.31, P = 0.033) were more likely to be seropositive. The ranges of measured visual acuity [6/5 to 6/60, equivalent to 20/17–20/200)] were similar in RVFV seropositive and seronegative groups, but visual acuity was more likely to be worse in the RVFV seropositive group (visual impairment defined as ≤20/80: 43% of seronegative vs. 80% of seropositive participants; *P*<0.0001).

## Discussion

This is the first cohort study performed on RVFV in a high-risk area of Kenya to document seroconversion and risk over time. This study demonstrates the significant RVFV seroprevalence (up to 33%) in an at-risk population in Northeastern Kenya, and highlights the differences in exposure between similar villages. Of the newly tested individuals who were randomly sampled, 29% were seropositive, highlighting the high risk of exposure in this region. Older age, rural village location, raw milk consumption, and poor visual acuity were significantly associated with RVFV seropositivity. We also documented the maintenance of PRNT_80_ titers at levels that are considered to be protective from disease over time in repeat participants.

RVFV seropositivity was relatively high in our sample population in Masalani town, Kenya, particularly in the rural village area (Gumarey), where seropositivity rates were twice as high as in the town area (Sogan-Godud) regardless of only 500 m of separation between the village sites. Our previous study also showed that those in Gumarey were at higher risk of seropositivity (20% vs. 6% in SG). Although seroprevalence in both villages has increased since 2006 (currently 33% vs. 15%), the differential between the two villages remains. Clues to the reasons for this discrepancy in seroprevalence were identified in our study. Those from Gumarey were significantly more likely to have particular kinds of animal exposures than those from Sogan-Godud. Village-specific models to predict seropositivity differ: age was the only independent predictor in Gumarey, suggesting that continued exposure over a life-time is the most important factor in this high risk village. In comparison, the model in SG highlighted both age and drinking raw milk, a factor that was important in the overall model for the entire study sample. Outbreak education may need to take village factors into account. As prediction tools are refined, better resolution may be needed to more accurately predict risk, since risk varies at such a small scale.

Our 2009 testing indicated that children had less evidence of past RVFV exposure than did adults in the same study cohort. This may be because children had less high-risk animal contact than adults. Alternatively, because we observed such an apparently low RVFV infection incidence among villagers in our study, it cannot be excluded that the study villages were not as heavily exposed to RVFV during the 2006–2007 outbreak as they had been in 1997–1998 RVFV outbreak. Because most children sampled in the present 2009 survey were born after the 1997–98 outbreak, if 2006–2007 transmission had been relatively low, their 2009 serostatus would be much more likely to remain negative in 2009.

PRNT_80_ titers remained high in individuals who were positive in the previous serosurvey, supporting previous expert opinion that development of anti-RVFV neutralizing titers through natural infection is likely to confer long-term protection against reinfection. Of note, one 54 y/o businessman had a boost in titer but did not report any signs of RVFV or animal contact in the last three years. He may have been re-exposed to RVFV, but suffered no clinical disease because of adaptive immunity persisting from a prior exposure.

In contrast to our previous studies, ELISA testing was not fully congruent with PRNT_80_. Ongoing repeat testing by PRNT_80_ will illustrate whether cross reactivity represented exposure to a concomitant circulating Bunyavirus.

Consumption of raw animal milk was associated with nearly 3 times the odds of RVFV seropositivity. Epidemiologic studies during RVF outbreaks have shown that drinking raw milk increases human risk for RVF disease, although whether the route of transmission is the direct consumption of infective raw milk or secondary to an alternate behavioral risk has not been determined [23], [24], [30], [31]. Analysis of milk products from experimentally infected animals provides conflicting evidence of virus infectivity in this body fluid [32]–[35] and attempts to infect offspring via suckling have failed to demonstrate transmission [2]. It is possible that the association that we detected between raw milk consumption and RVFV seropositivity is not causal and may represent some unmeasured variable, although history of milking livestock was not associated with RVFV seropositivity. Laboratory based experiments to determine the viability and transmissibility of RVFV in milk are warranted.

The most common sequela of RVFV infection is uveitis. Persons who were RVFV seropositive were more likely to have poor visual acuity, but a large portion of the study sample had poor eyesight. RVFV is one of many eye diseases present in Kenya. It is likely that RVFV along with trachoma, West Nile virus, chikungunya virus, dengue virus, and others all contribute to the significant burden of poor vision in our cohort.

Apart from eye disease, no physical examination finding was associated with RVFV seropositivity, although several RVFV-associated symptoms were reported among those who were RVFV seropositive. Many were severe manifestations of RVF disease, such as bloody stools, confusion, and stupor. Confusion and stupor may represent those with history of RVF encephalitis. Malaise, backache, and rash may represent those with history of mild RVF or other illnesses.

Our study was limited by its small sample size; although three attempts were made to re-enroll our 248 previous participants, only 102 were studied. Because only one individual seroconverted we cannot draw conclusions about whether the identified risk factors specifically caused RVFV exposure. Reported associated symptoms may have been due to other infections; for example, RVF is usually not associated with rash. Our study had a larger proportion of women, since the men in this community are often herding and may not be near the homestead. This bias in our study sample may underestimate the incidence and prevalence of RVFV exposure in this community, since males are more at risk. The validity of the associations in this study relies on accurate recall of exposures by the study participants and our study may have limited generalizability.

This study highlights the high seroprevalence among Northeastern Kenyans and the ongoing surge in seroprevalence with each RVF outbreak. Consumption of raw milk may be an easy target for effective prevention of RVF during outbreaks and warrants further study. Local public health agencies may need to target specific protective interventions according to risk factors in different populations.

## Supporting Information

Appendix S1 **Questionnaire and data entry forms used in survey.**
(PDF)

Table S1 **Logistic Regression Analysis to predict Rift Valley fever virus seropositivity-by participant group.** Table S1A: Logistic Regression Analysis to predict Rift Valley fever virus seropositivity-new participants only*. CI, 95% confidence interval. Goodness-of-fit: Hosmer and Lemeshow test, p value = 1.0, R^2^ = 14%. Table S1B: Logistic Regression Analysis to predict Rift Valley fever virus seropositivity-repeat participants only*. * CI, 95% confidence interval. Goodness-of-fit: Hosmer and Lemeshow test, p value = 0.338, R^2^ = 14%.
(DOCX)

Table S2 **Logistic Regression Analysis to predict Rift Valley fever virus seropositivity-by village.** Table S2A: Logistic Regression Analysis to predict Rift Valley fever virus seropositivity-Sogan-Godud residents only*. * CI, 95% confidence interval. Goodness-of-fit: Hosmer and Lemeshow test, p value = 0.153, R^2^ = 33%. Table S2B: Logistic Regression Analysis to predict Rift Valley fever virus seropositivity-Gumarey residents only*. * CI, 95% confidence interval. Goodness-of-fit: Hosmer and Lemeshow test, p value = 0.304, R^2^ = 23%.
(DOCX)
