# Supplementary material for: Postepidemic Analysis of Rift Valley Fever Virus Transmission in Northeastern Kenya: A Village Cohort Study
Source: PLoS Negl Trop Dis. 2011 Aug 16;5(8):e1265. doi: 10.1371/journal.pntd.0001265 (PMC3156691; doi:10.1371/journal.pntd.0001265)
Supplement: Appendix S1 — Questionnaire and data entry forms used in survey. (PDF) [file pntd.0001265.s001.pdf]

Viral Causes of Severe Febrile Illness: PERSONAL DETAILS

ID: 1

Date of Registration (dd/mm/yyyy):

Subject's First Name:

Second Name:

Third Name:

Other name:

House Number (DSS):

Village:

District:

Province:

Head of Household's Name:

Relationship to HH:

Usual Occupation:

Do you SLEEP at this house? ☐

Do you STAY at this house? ☐

Year of Birth:

Sex (0=Female, 1=Male):

Age in years:

School:

Moved Here in last 2 yrs? ☐

Permanent Village Resident? ☐

Semi-Nomadic? ☐

When?:

From Where?:

Past Medical History:

Current Medicines:

Assistant Information

Name of Data Collector:

Name of Data Enterer:

Viral Causes of Severe Febrile Illness: PATIENT SYMPTOMS

ID 1

How long have you been sick?:

Have you had any of these symptoms since your illness began?

Fever ☐	Chills ☐	Flushing ☐
Sick Feeling ☐	No appetite ☐	
Backache ☐	Muscle aches ☐	
Nausea ☐	Vomiting ☐	Vomiting Blood ☐
Bloody stool ☐		
Rash ☐	Bruising ☐	Nosebleeds ☐
Headache ☐	Neck stiffness ☐	
Red eyes ☐	Eye pain ☐	Painful eyes to light ☐
Poor vision ☐		
Spinning feeling ☐	Confusion ☐	
Hard to arouse ☐	Coma ☐	

What is the patient's Clinical Syndrome best described as?

Principal Investigator: A. Desiree LaBeaud

Viral Causes of Severe Febrile Illness: NON-ANIMAL EXPOSURES

ID 1 Type of settlement

Home flooded? ☐ When was it flooded?

Displacement by flood? ☐ When were you displaced?

Do you use mosquito net? ☐ How often use net?

Indicate: Never, Other, Monthly, Weekly, or Daily

Do you use fire? ☐ How often do you use fire?

Indicate: Never, Other, Monthly, Weekly, or Daily

Do you use mosquito coil? ☐ How often coils?

Indicate: Never, Other, Monthly, Weekly, or Daily

Do you use other mosquito control? What kind?

Do you have screens on your home windows? ☐

When was the last time you had a mosquito bite?

Indicate: < 1month, 1-3 mo, 4-6mo, 7-12mo, 1-2 yr, or never

When was the last time you were ill?

Indicate: < 1month, 1-3 mo, 4-6mo, 7-12mo, 1-2 yr, or never

When did you last have an ill family member?

Indicate: < 1month, 1-3 mo, 4-6mo, 7-12mo, 1-2 yr, or never

When was your last contact with a dead human body?

Indicate: < 1month, 1-3 mo, 4-6mo, 7-12mo, 1-2 yr, or never

What type of roofing do you have in your home?

Indicate: natural material, corrugated metal or plastic, or other

Which would best describe your latrine?

Indicate: bush, pit, VIP latrine, toilet, other

What type of flooring do you have in your home?

Indicate: dirt, wood, cement, tile, or other

Where does your drinking water come from?

Indicate: river/pond, rain, public well/borehole, inside well, public tap/piped, or other

Are their objects around your home that collect water? ☐

Please list the objects.

Do mosquitoes bother you during the day time? ☐

Do mosquitoes bother you during the night time? ☐

How much time do you spend outdoors each day?:

How often are you bitten by mosquitoes:

Do you avoid mosquitoes? ☐

Principal Investigator: A. Desiree LaBeaud

Viral Causes of Severe Febrile Illness: ANIMAL EXPOSURES

ID 1

How often do you have sheep contact?: ☐

Indicate: Never, Other, Monthly, Weekly, or Daily

How often do you have goat contact?: ☐

Indicate: Never, Other, Monthly, Weekly, or Daily

How often do you have cow contact?: ☐

Indicate: Never, Other, Monthly, Weekly, or Daily

How often do you have camel contact?: ☐

Indicate: Never, Other, Monthly, Weekly, or Daily

Have you SHELTERED livestock in your home? ☐

camel ☐ sheep ☐ goat ☐ cow ☐ other ☐

Have you KILLED an animal? ☐

camel ☐ sheep ☐ goat ☐ cow ☐ other ☐

Have you BUTCHERED an animal? ☐

camel ☐ sheep ☐ goat ☐ cow ☐ other ☐

Have you SKINNED an animal? ☐

camel ☐ sheep ☐ goat ☐ cow ☐ other ☐

Have you COOKED WITH MEAT? ☐

camel ☐ sheep ☐ goat ☐ cow ☐ other ☐

Have you MILKED an animal? ☐

camel ☐ sheep ☐ goat ☐ cow ☐ other ☐

Have you DRUNK RAW MILK? ☐

camel ☐ sheep ☐ goat ☐ cow ☐ other ☐

Have you CARED FOR A BIRTHING ANIMAL? ☐

camel ☐ sheep ☐ goat ☐ cow ☐ other ☐

Have you DISPOSED OF AN ABORTED ANIMAL FETUS? ☐

camel ☐ sheep ☐ goat ☐ cow ☐ other ☐

Viral Causes of Severe Febrile Illness: PHYSICAL EXAM

ID 1 **Weight in kg** ____ **Height in cm** ____

General Wasted? ☐ ____

Head ____

Eyes ____

Scleral hemorrhages? ☐ Scleral Icterus? ☐

Ears ____

Nose ____

Throat ____

Neck ____

Normal movement? ☐

Chest ____

Heart ____

Murmur? ☐

Abdomen ____

Hepatomegaly? ☐ Splenomegaly? ☐

GU ____

Neuro ____

Skin ____

Jaundice? ☐ Petechiae? ☐ Purpura? ☐ Ecchymosis? ☐

Lymphadenopathy ____

Cervical ☐ Axillary ☐ Inguinal ☐

Other ____

Name of Medical Doctor ____

Name of Data Enterer ____

Principal Investigator: A. Desiree LaBeaud

Viral Causes of Severe Febrile Inness: OPHTHALMOLOGIC EXAM

ID 1

Visual Acuity-OS

Visual Acuity-OD

Anterior Chamber-OS

Anterior Chamber-OD

Anterior Uveitis-OS? ☐

Anterior Uveitis-OD? ☐

Posterior Chamber-OS

Posterior Chamber-OD

Vitreous reaction-OS? ☐

Vitreous reaction-OD? ☐

Retina-OS

Retina-OD

Retinitis-OS? ☐ Macular-OS ○ Paramacular-OS ○

Retinitis-OD? ☐ Macular-OD ○ Paramacular-OD ○

Retinal Hemorrhage-OS? ☐

Zone-OS

Area-OS

Retinal Hemorrhage-OD? ☐

Zone-OD

Area-OD

Optic disc edema-OS? ☐

Optic disc edema-OD? ☐

Retinal vasculitis-OS? ☐

Retinal vasculitis-OD? ☐

RVF Related Disease-OS? ☐

RVF Related Disease-OD? ☐

Comments

Ophthalmologist Name

Data Enterer Name
