# Supplementary material for: Postepidemic Analysis of Rift Valley Fever Virus Transmission in Northeastern Kenya: A Village Cohort Study
Source: PLoS Negl Trop Dis. 2011 Aug 16;5(8):e1265. doi: 10.1371/journal.pntd.0001265 (PMC3156691; doi:10.1371/journal.pntd.0001265)
Supplement: Table S1 — Logistic Regression Analysis to predict Rift Valley fever virus seropositivity-by participant group. Table S1A: Logistic Regression Analysis to predict Rift Valley fever virus seropositivity-new participants only*. CI, 95% confidence interval. Goodness-of-fit: Hosmer and Lemeshow test, p value = 1.0, R2 = 14%. Table S1B: Logistic Regression Analysis to predict Rift Valley fever virus seropositivity-repeat participants only*. * CI, 95% confidence interval. Goodness-of-fit: Hosmer and Lemeshow test, p value = 0.338, R2 = 14%. (DOCX) [file pntd.0001265.s002.docx]

**Table S1A: Logistic Regression Analysis to Predict Rift Valley fever virus seropositivity-New Participants Only***

| **Predictor variable** | **Variable type** | **Adjusted OR (CI)** | ***P* value** |
| --- | --- | --- | --- |
| Assist with birthing animal | Dichotomous | 7.2 (1.9–26.7) | 0.003 |

* CI, 95% confidence interval. Goodness-of-fit: Hosmer and Lemeshow test, p value = 1.0, R^2^ = 14%.

**Table S1B:** **Logistic Regression Analysis to Predict Rift Valley fever virus seropositivity-Repeat Participants Only***

| **Predictor variable** | **Variable type** | **Adjusted OR (CI)** | ***P* value** |
| --- | --- | --- | --- |
| Age | Continuous | 1.05 (1.02–1.07) | <0.0001 |
| Gender (female vs. male) | Dichotomous | 0.25 (0.07–0.81) | 0.017 |

* CI, 95% confidence interval. Goodness-of-fit: Hosmer and Lemeshow test, p value = 0.338, R^2^ = 14%.
