# Supplementary material for: Postepidemic Analysis of Rift Valley Fever Virus Transmission in Northeastern Kenya: A Village Cohort Study
Source: PLoS Negl Trop Dis. 2011 Aug 16;5(8):e1265. doi: 10.1371/journal.pntd.0001265 (PMC3156691; doi:10.1371/journal.pntd.0001265)
Supplement: Table S2 — Logistic Regression Analysis to predict Rift Valley fever virus seropositivity-by village. Table S2A: Logistic Regression Analysis to predict Rift Valley fever virus seropositivity-Sogan-Godud residents only*. * CI, 95% confidence interval. Goodness-of-fit: Hosmer and Lemeshow test, p value = 0.153, R2 = 33%. Table S2B: Logistic Regression Analysis to predict Rift Valley fever virus seropositivity-Gumarey residents only*. * CI, 95% confidence interval. Goodness-of-fit: Hosmer and Lemeshow test, p value = 0.304, R2 = 23%. (DOCX) [file pntd.0001265.s003.docx]

**Table S2A: Logistic Regression Analysis to Predict Rift Valley fever virus seropositivity-Sogan Gudud Residents Only***

| **Predictor variables** | **Variable type** | **Point estimate (CI)** | ***P* value** |
| --- | --- | --- | --- |
| Age | Continuous | 1.05 (1.02–1.07) | <0.0001 |
| Cooked meat | Dichotomous | 0.184 (0.42–0.810) | 0.025 |
| Drank raw milk | Dichotomous | 15.7 (2.9–84.9) | 0.001 |

* CI, 95% confidence interval. Goodness-of-fit: Hosmer and Lemeshow test, p value = 0.153, R^2^ = 33%.

**Table S2B: Logistic Regression Analysis to Predict Rift Valley fever virus seropositivity-Gumarey Residents Only***

| **Predictor variables** | **Variable type** | **Point estimate (CI)** | ***P* value** |
| --- | --- | --- | --- |
| Age | Continuous | 1.05 (1.02–1.07) | 0.001 |

* CI, 95% confidence interval. Goodness-of-fit: Hosmer and Lemeshow test, p value = 0.304, R^2^ = 23%.
